# Using bibliometrics to evaluate translational science training: evidence for early career success of KL2 scholars

**DOI:** 10.1017/cts.2020.516

**Published:** 2020-07-24

**Authors:** Kelli Qua, Clara M. Pelfrey

**Affiliations:** School of Medicine, Clinical and Translational Science Collaborative, Case Western Reserve University, Cleveland, OH, USA

**Keywords:** Bibliometrics, Clinical and Translational Science Award, translational research, KL2, career development

## Abstract

**Introduction:**

Evaluating clinical and translational research (CTR) mentored training programs is challenging because no two programs are alike. Careful selection of appropriate metrics is required to make valid comparisons between individuals and between programs. The KL2 program provides mentored-training for early-stage CTR investigators. Clinical and Translational Awards across the country have unique KL2 programs. The evaluation of KL2 programs has begun to incorporate bibliometrics to measure KL2 scholar and program impact.

**Methods:**

This study investigated demographic differences in bibliometric performance and post-K award funding of KL2 scholars and compared the bibliometric performance and post-K award federal funding of KL2 scholars and other mentored-K awardees at the same institution. Data for this study included SciVal and iCite bibliometrics and National Institutions of Health RePORTER grant information for mentored-K awardees (K08, K23, and KL2) at Case Western Reserve University between 2005 and 2013.

**Results:**

Results showed no demographics differences within the KL2 program scholars. Bibliometric differences between KL2 and other mentored-K awardee indicated an initial KL2 advantage for the number of publications at 5 years’ post-matriculation (i.e., the start of the K award). Regression analyses indicated the number of initial publications was a significant predictor of federal grant funding at the same time point. Analysis beyond the 5-year post-matriculation point did not result in a sustained, significant KL2 advantage.

**Conclusions:**

Factors that contributed to the grant funding advantage need to be determined. Additionally, differences between translational and clinical bibliometrics must be interpreted with caution, and appropriate metrics for translational science must be established.

## Introduction

Rigorous evaluation of a clinical and translational research (CTR) training program requires careful selection of reliable quantitative metrics and matching to a reasonable comparison group. This study expands evaluation methods to assess the KL2 program. The KL2 program is a multiyear mentored training award focused on career development for early-stage CTR investigators. The KL2 program is a workforce development component of the larger Clinical and Translational Award (CSTA) granted by the National Center for Advancing Translational Sciences (NCATS) at the National Institutions of Health (NIH). Over 60 funded CSTAs across the country have KL2 programs and no two are alike. While the programs vary greatly, they do include several overlapping characteristics such as interdisciplinary mentorship, training opportunities aligned to CTR competencies [1] such as team science, scientific and grant writing, career development planning, and protected research time. Outside of these programmatic requirements that are standard across Clinical and Translational Science Awards (CTSAs), one recent study investigating KL2 program differences surveyed 55 CSTA hubs (90%) and reported that 87% of KL2 scholars had 2–3 years of supported (i.e., funded) KL2 training [2]; at least one CTSA provides 4 years of funding. The variability in length of funding is just one example of the various KL2 program differences, which make a standardized evaluation difficult.

The extent of program-level differences is also present when considering the outcome measures of the KL2 program across the CSTAs. While the primary outcome across KL2 programs is to establish independent CTR investigators, institutions operationalize “independent funding” differently. Some examples of the variability in the definition of achieving independent funding include: principal investigator (PI) roles on any grant, PI on an NIH R01 grant, other key roles such as Co-PI or Co-I on an NIH R01 grant, R-level funding (either NIH or Non-NIH), and time to R01 [3–5]. Independent funding, in any of these forms, may be the primary outcome for KL2 programs, but it is not the singular outcome. When KL2 programs only use independent grant funding as a measure of KL2 program success, they fail to account for the more intermediate outcomes that impact funding such a publications, collaborations, and smaller foundation and/or pilot grant awards, for example.

Recently, the evaluation of KL2 programs has looked to bibliometrics (i.e., the science and quantification of publication data) to provide a broader picture of KL2 scholar and program impact. Several methods for using bibliometrics in this capacity have been tested for their efficacy, such as comparisons of paid services (e.g., Elsevier products) and the use of open-source tools (e.g., iCite) [6]. The strengths of these bibliometric methods are that they provide insight into a more diverse set of research outcomes, including collaboration, dissemination patterns, citation and publication networks, and publication impact [7]. Advanced bibliometric methods can facilitate the evaluation of unique KL2 programs by expanding the definition of research productivity [8].

In an effort to expand the existing evaluation efforts conducted by other CTSAs, this study aimed to explore the bibliometric outcomes and federal follow-on funding of KL2 scholars in comparison to other K scholars at the same institution in order to assess the impact of the KL2 program on research outcomes. The present research had three objectives: (1) to investigate whether there were demographic differences (e.g., gender, race, and degree) in bibliometric performance or post-K award federal grant funding of KL2 scholars, (2) to compare the bibliometric performance between KL2 scholars and other to other mentored-K awardees (K08 and K23), and (3) to compare post-K award federal grant funding of KL2 scholars compared to other mentored-K awardees (K08 and K23).

## Methods

### Participants

Early-stage investigators who were awarded NIH clinical, translational, or behavioral mentored-K awards (K08, K23, KL2) at Case Western Reserve University (CWRU) between 2005 and 2013 were included in this analysis. K08 and K23 mechanisms were selected as a comparison group because they are similar to the KL2 mechanism in that (1) these mechanisms provide protected time for early-stage investigators and (2) they focus on scholars in “biomedical and behavioral research, including translational research” and “patient-oriented research” [9]. At CWRU, the K08 and K23 awards differ from the KL2 in that K08 and K23 scholars are not required to take coursework, have different funding lengths (i.e., the KL2 program studied here provides 4 years of funding; the K08/K23 scholars reported between three to 5 years of funding, with an average funding length of 4.5 years), and typically focus on clinical research. Other NIH career development K awards exist (e.g., K01, K25, K99), but these K awards do not specifically target early-stage CTR and/or behavioral investigators.

Participants were selected using the date range of 2005–2013 to ensure all scholars had a minimum of 5 years of data from the start of the K award (i.e., matriculation). Thirteen K12 scholars (NIH institutional career development awards) from 2005 to 2006 were transitioned to KL2 scholars when the institution’s CTSA was initially awarded in 2007. Demographics of the K12s from 2005 to 2006 and KL2 scholars are comparable.

### Measures

A series of bibliometrics were investigated in order to produce a valid and reliable data set. One set of metrics was exported from the Elsevier’s SciVal [10] database, and a second set of metrics was exported from the NIH’s iCite [11] database. Extracting information from both of these databases reduces possible bias and provides a more reliable estimate of the quantity and impact of scholarly work. iCite is limited to analyzing only articles that appear in PubMed and is a public and government-verified source, whereas SciVal uses publication information from the more comprehensive Scopus [12] database.

Bibliometrics exported from SciVal were chosen to provide evidence for three domains of bibliometrics: productivity, impact, and collaboration. A detailed list of metrics is presented in Table 1. Productivity metrics provide an overview of total scholarly output; in other words, how many publications a scholar produces within a time period. Impact metrics focus on citation count through raw and field-weighted or ratio values. Self-citations were excluded from analysis. Collaboration metrics consider all authors on a scholar’s publication with attention to co-authors’ affiliated institutions. These metrics represent both author-level and publication-level data. Author-level metrics, such as total number of publications, are those which directly quantify the output of the author, providing an overview of the author’s overall publication portfolio. Publication- or article-level metrics, such as citation rates, are representative of the performance of an individual publication. However, some article-level metrics when aggregated or normed, such as the number of citations for one publication, may show author-level information such as the author’s average number of citations which encompasses all publications. Information on the level of the metrics used in this study is denoted in Table 1.

**Table 1.** Summary of metrics used for analysis

Source	Domain	Metric	Definition
SciVal	Productivity	Scholarly output+	The number of publications indexed in Scopus
	Impact	Citations per publication*	The average number of citations received per publication**
		% of publications in the top 10th percentile of journals*	The number of publications in the world’s top journals***
		Field-weighted citation impact+	The ratio of citations received relative to the expected world average for the subject field, publication type, and publication year.
	Collaboration	National, International, Institutional*	Each publication is assigned to one of four mutually exclusive collaboration types, based on affiliation information: international, national, institutional, or single authorship. A single publication may display each collaboration type, but a single collaboration type is assigned to ensure that the sum of an entity’s publications across the four categories adds up to 100% of the publications with the necessary affiliation information.
		National field-weighted+	The collaboration ratio is computed based on the expected collaboration for that document type, publication year grouping, and subject area assignment.
iCite	Productivity	Total number of publications+	The number of publications indexed in iCite
	Impact	Average RCR+ [14]	The cites/year of each paper, normalized to the citations per year received by NIH-funded papers in the same field and year
		Weighted RCR+	The number of articles multiplied by their average RCR

* Represents a publication or article-level metric.

+ Represents an author-level metric – note many metrics can be article level but were analyzed in the aggregate or as normed-values of author-level data for this study.

** Self-citations were excluded from this analysis.

*** The most-cited journals are defined by the journal metrics CiteScore, Source-Normalized Impact per Paper, or SCImago Journal Rank.

RCR, relative citation ratio.

Three measures of grant funding were also collected: NIH funding as PI, Co-PI, Co-Investigator at the 5 year, 8 year, and overall time points. These measures of follow-on funding include only NIH federal funding received by a scholar identified as participating in the KL2, K08, or K23 program at CWRU from 2005 to 2013. Each of the three measures is a dichotomous variable of NIH federal funding received. Records of NIH funding can be found on NIH RePORTER [13].

### Procedures

This study received institutional review board (IRB) exemption (IRB #20190435). The research team contacted the Office of Grants and Contracts for a list of all mentored-K award scholars at the institution since 2005. A librarian identified author IDs from Scopus/SciVal and provided SciVal training to the research team. Cohorts were created in SciVal based on the type of K award (KL2 or K08/K23) and the year the scholar began their K-grant (i.e., all 2005 KL2’s represent one cohort). All metrics from SciVal and iCite were extracted and entered into an SPSS file within a one month period. Grant data were collected using records of federal funding which were confirmed through NIH Reporter.

### Analysis

Statistics were computed using IBM’s SPSS version 25 (IBM Corp., Armonk, NY, USA). Due to the variability of these data, there were several outliers for each metric. Non-parametric tests (Mann–Whitney U) were used when data were not normally distributed and medians (*Mdn*) and interquartile ranges (*IQR*) provided. Descriptive and inferential statistics were used to analyze the bibliometrics of KL2 scholars according to demographic groups and the bibliometric performance of KL2 scholars and K08/K23 scholars at the same institution. Federal funding data were analyzed using logistic rgression to assess the relationship between any significant variables found in the bibliometric analysis and a series of dichotomous funding variables.

Data were analyzed at three time points after matriculation: 5 years, 8 years [15,16], and overall (i.e., from the start year of a scholar’s K award through the end of 2018, regardless of the number of years). All data were time bound to include the year the scholar started their K award through the completion of 2018. iCite data were not included for the 5- and 8-year time points. This is because the main iCite metric, the relative citation ratio, is subject to latency, which means that the metric needs more time to develop and is not accurate for “new” publications [17]. One KL2 scholar and one K08 were excluded from analyses due to lack of data. The 8-year matriculation analysis included only scholars who began their K award in 2011 or earlier.

## Results

Eighty-five K Scholars met the criteria for analyses across the K08, K23, and KL2 grant mechanisms. K08 and K23 scholars were collapsed into one group for analysis to increase sample size equivalence. Additionally, combining the K08 and K23 scholars into one group more accurately matches the KL2 scholars’ research domains, which include behavioral, translational, and clinical research. Differences in the demographic composition of groups were analyzed. There was a significant difference between scholar degrees, with KL2 scholars holding more PhDs and the K08/K23 scholars more likely to have MDs or MD/PhDs (*X*2(12) = 27.59; *p* = .006). However, the majority of scholars in both groups held MDs. A summary of demographic information for KL2 and K08/K23 scholars is reported in Table 2.

**Table 2.** Summary of scholar demographics

Demographics	Sample (*N* = 85)	KL2 (*n* = 50)	K08/K23 (*n* = 35)
*Gender*			
Male	**56% (48)**	**54% (27)**	**60% (21)**
Female	44% (37)	46% (23)	40% (14)
*Ethnicity*			
Hispanic/Latino	4.7% (4)	8% (4)	0% (0)
Not Hispanic/Latino	**93% (79)**	**90% (45)**	**97% (34)**
Not reported	2.3 % (2)	2% (1)	3% (1)
*Race*			
American Indian/Alaskan	1.1% (1)	0% (0)	2.9% (1)
Asian	23.5% (20)	18% (9)	31.4% (11)
African American	3.5% (3)	6% (3)	0% (0)
Caucasian	**68.2% (58)**	**72% (36)**	**62.8% (22)**
Not reported	3.5% (3)	4% (2)	2.9% (1)
*Degree*			
DDS	1.1% (1)	2% (1)	0% (0)
MD	**51.7% (44)**	**40% (20)**	**68.5% (24)**
PhD	20% (17)	34% (17)	0% (0)
RN, PhD	4.7% (4)	8% (4)	0% (0)
Other	1.1% (1)	0% (0)	2.8% (1)
MD, PhD	20% (17)	14% (7)	28.5% (10)
DO, PhD	1.1% (1)	2% (1)	0% (0)
*Year*			
2005	**20% (17)**	**16% (8)**	**25.7% (9)**
2006	11.7% (10)	10% (5)	14.2% (5)
2007	14.1% (12)	10% (5)	20% (7)
2008*	9.4% (8)	12% (6)	5.7% (2)
2009	8.2% (7)	10% (5)	5.7% (2)
2010	10.5% (9)	8% (4)	14.2% (5)
2011	7.1% (6)	8% (4)	5.7% (2)
2012*	8.2% (7)	10% (5)	5.7% (2)
2013	10.5% (9)	**16% (8)**	2.8% (1)

* Indicates a cohort with a scholar excluded from analysis.

*Note*: One KL2 scholar from 2008 and one K08 scholar from 2012 were excluded from analysis due to lack of data. Bold text indicates the highest value.

**Research Question 1:** Are there demographic differences (gender, race, and degree) in bibliometric performance of KL2 scholars?

There were no significant differences in productivity, impact, or collaboration between male and female KL2 scholars, between Caucasian and Asian/African American KL2 scholars, or between scholars with clinical degrees (i.e., MDs) and those with clinical training and/or other professional/doctoral degrees (i.e., PhDs) at any time point. Ethnicity was not analyzed as the composition of the sample was 93% White, Non-Hispanic/Latino. No analyses between cohorts (e.g., the 2005 cohort and the 2006 cohort) were conducted due to the limited sample size per cohort (data not shown).

**Research Question 2:** Is there a difference between the bibliometric performance of KL2 scholars compared to other mentored-K awardees (K08 and K23) at the same institution?

Five years after matriculation, there were significant differences between KL2 and K08/K23 scholars on one productivity metric and one collaboration metric (Table 3). Examining scholarly output, KL2 scholars (*Mdn* = 15; *IQR* = 21.50) published significantly more than K08/K23 scholars (*Mdn* = 10; *IQR* = 10.50) during these 5 years (U = 608.50; *p* = .03). Measuring collaboration, KL2 scholars (*M* = .75; *SD* = .45) demonstrated higher field-weighted collaborations at the national level (*t*(81) = 2.12; *p* = .03) than K08/K23 scholars (*M* = .55; *SD* = .37).

**Table 3.** Results of 5-Year Metrics for KL2 and K08/K23 Scholars (*N* = 83)

Domain	Metric	KL2 (*n* = 49)	KO8/K23 (*n* = 34)	*P* value
Productivity	Scholarly output	***Mdn*** = **15**	*Mdn* = 10	***p*** = .03*
Impact	Citations per publication	*Mdn* = 93.91	*Mdn* = 139.50	*p* = .054
	% of publications in the top 10th percentile of journals	*M* = 38.13%	*M* = 42.99%	*p* = .34
	Field-weighted citation impact	*Mdn* = 6.79	*Mdn* = 6.32	*p* = .77
Collaboration	National	*M* = 37.77	*M* = 29.62	*p* = .11
	National field-weighted	***M*** = **.75**	*M* = .55	***p*** = .03*
	International	*Mdn* = 9.68	*Mdn* = 12.00	*p* = .40
	Institutional	*Mdn* = 25.14	*Mdn* = 30.00	*p* = .50

*p < .05

*Note*: To indicate descriptive statistics, some notation was used: *Mdn* (median), *M* (mean), were reported for non-normal distributions. Bold text indicates the highest value.

An analysis of KL2 and K08/K23 scholars with 8 years of bibliometric data after matriculation again revealed significantly higher scholarly output (*U* = 412.50; *p* = .04) in KL2 scholars (*Mdn* = 27; *IQR* = 34.25) than in K08/K23 scholars (*Mdn* = 20; *IQR* = 20). In contrast, 8 years after matriculation, K08/K23 scholars (*Mdn* = 19.23; *IQR* = 19.59) had more international collaborations (*U* = 392.50; *p* = .024) than KL2 scholars (*Mdn* = 8.33; *IQR* = 22.39) (Table 4).

**Table 4.** Results of 8-Year Metrics for KL2 and K08/K23 Scholars (*N* = 63)

Domain	Metric	KL2 (*n* = 36)	KO8/K23 (*n* = 32)	*P* value
Productivity	Scholarly output	***Mdn*** = **27**	*Mdn* = 20	***p*** = .04*
Impact	Citations per publication	*Mdn* = 161.30	*Mdn* = 254.65	*p* = .11
	% of publications in the top 10th percentile of journals	*M* = 37.79%	*M* = 45.42%	*p* = .22
	Field-weighted citation impact	*Mdn* = 10.16	*Mdn* = 11.57	*p* = .572
Collaboration	National	*M* = 35.66	*M* = 29.35	*p* = .104
	National field-weighted	*M* = .69	*M* = .56	*p* = .084
	International	*Mdn* = 8.33	***Mdn*** = **19.23**	***p*** = .024*
	Institutional	*M* = 31.51	*M* = 29.86	*p* = .539

*p < .05

*Note*: To indicate descriptive statistics, some notation was used: *Mdn* (median), *M* (mean), were reported for non-normal distributions. Bold text indicates the highest value.

Analyses of the overall time point (i.e., all years from matriculation to the end of 2018) is reported in Table 5. In contrast to the 5- and 8-year metrics, overall (from the year a scholar started a K award through year end of 2018), there were no significant differences between KL2 and K08/K23 scholars in scholarly output. There was a significant difference between KL2 and K08/K23 scholars on two impact metrics. Overall, K08/K23 scholars (*Mdn* = 135.9; *IQR* = 26.33) had significantly more citations per publication (*U* = 609; *p* = .018) than KL2 scholars (*Mdn* = 268.20; *IQR* = 13.90), and K08/K23 scholars (*M* = 51.49; *SD* = 19.8) were published more in top percentile journals (*t*(81) = 2.255; *p* = .027) than KL2 scholars (*M* = 41.81; *SD* = 18.84). There was a significant difference between KL2 and K08/K23 scholars on two collaboration metrics. Overall, K08/K23 scholars (*Mdn* = 24.95; *IQR* = 21.57) had higher international collaboration (*U* = 618.50; *p* = .047) than KL2 scholars (*Mdn* = 12.80; *IQR* = 26.30), but KL2 scholars (*M* = .86; *SD* = .36) had a higher field-weighted national collaboration (*t*(81) = 2.122; *p* = .037) than K08/K23 scholars (*M* = .77; *SD* = .36).

**Table 5.** Results of Overall Metric for KL2 and K08/K23 Scholars (*N* = 83)

Domain	Metric	KL2 (*n* = 33)	KO8/K23 (*n* = 30)	*P* value
Productivity	Scholarly output	*Mdn* = 40.50	*Mdn* = 27.50	*p* = .115
	Total number of publications	*Mdn* = 26.50	*Mdn* = 26.00	*p* = .566
Impact	Citations per publication	*Mdn* = 135.9	***Mdn*** = **268.2**	***p*** = .018*
	% of publications in the top 10th percentile of journals	*M* = 41.81%	***M*** = **51.49%**	***p*** = .027*
	Field-weighted citation impact	*Mdn* = 1.59	*Mdn* = 1.72	*p* = .362
	Average RCR	*Mdn* = 1.53	*Mdn* = 1.59	*p* = .381
	Weighted RCR	*Mdn* = 31.13	*Mdn* = 34.70	*p* = .882
Collaboration	National	*M* = 42.61	*M* = 37.36	*p* = .343
	National field-weighted	***M*** = **.86**	*M* = .77	***p*** = .037*
	International	*Mdn* = 12.80	***Mdn*** = **24.95**	***p*** = .047*
	Institutional	*M* = 31.22	*M* = 34.83	*p* = .483

* *p* < .05

*Note*: To indicate descriptive statistics, some notation was used: *Mdn* (median), *M* (mean), were reported for non-normal distributions. Bold text indicates the highest value.

RCR, relative citation ratio.

**Research Question 3:** Is there a difference between post-K award federal grant funding (i.e., NIH) of KL2 scholars compared to other mentored-K awardees (K08 and K23) at the same institution?

Descriptive statistics indicated that 5 years after matriculation 32% of KL2 scholars and 31% of K08/K23 scholars had received NIH funding. NIH funding rates at the 8-year time point revealed that 48% of KL2s and 51% of K08/K23 scholars received NIH funding. To inform regression analyses, Pearson correlations were run using the significant variables found in the above inferential tests and grant funding outcomes. Several bibliometric variables were significantly positively correlated with NIH funding; however, the majority of the correlations were weak (<.03). Scholarly output (i.e., number of publications) was the strongest correlating variable across all time points for both KL2 and K08/K23 scholars (*r* = .3). Logistic Regression analyses reported that 5-year scholarly output significantly predicted KL2 funding outcomes (*X*2(1) = 5.26; *p* = .03; Nagelkerke *R*2 = 14%), but was non-significant for K08/K23 scholars (*X*2(1) = 2.61; *p* = .12). Overall total scholarly output (i.e., all years) reported no significant prediction for KL2 funding (*X*2(1) = 4.02; *p* = .06), but was significant for K08/K23 funding (*X*2(1) = 6.93; *p* = .03; Nagelkerke *R*2 = 24%).

## Discussion

The results of this analysis suggest that the KL2 scholars have an advantage over K08 and K23 scholars at the 5-year time point in regards to NIH funding and scholarly output, as measured by bibliometric performance. This advantage is largely driven by the number of publications KL2s have and their field-weighted collaborations, when compared to the other K scholars at the same institution. Over time, these bibliometric measures shift and indicate little difference between the two groups, but with K08/K23s reporting more international collaborations and publications in top-tier journals.

It is important to focus on the 5-year time point and the factors that may have influence on the NIH funding rate. The relationship between publications and funding has been investigated for decades in relation to return on investment [18–20]. In other words, funders consider if the investigator will publish the results of their project and evidence for this is a strong publication record prior to funding. KL2 scholars are able to provide a “larger” publication history before their K08/K23 counterparts. This is consistent with some previous research which suggests that K awards who published nine publications per K year were significantly more likely to convert to R-funding compared to those with four publications [5]; however, this research has thus far focused on K awardees who are surgeons. Additional research into the predictive relationship between publications and R-funding is needed for other early-career CTR investigators, such as KL2s.

This study’s findings indicating the initial high volume of KL2 publications may also be a reflection of the KL2 program’s structure and time for scholars to develop pilot data, which can be used in publications and grant applications. This means that immediately upon completion of the K-grant, the KL2s have a more established research presence (i.e., publications and pilot data), which initially leads to more funding. However, this initial advantage appears to diminish over time. Investigation into publication patterns and citation rates for clinicians versus non-clinician scientists should be explored. Further research into the factors that contribute to continued NIH funding for K-scholars should be considered.

The implications of using bibliometrics to evaluate translational research must also be considered by the research and evaluation community. In order to discuss this, we must first consider the definition of translational research. Understanding the difference between clinical and translational studies will help CSTAs and evaluators make informed decisions about how they evaluate scholarly performance when using publication data. The NIH defines clinical research as (1) patient-oriented with direct interaction with human subjects, (2) epidemiology and/or behavioral, or (3) outcomes or health services research [21]. The NIH definition of translational research is less precise and does not have clearly defined categories [22]; however, some translational science evaluators have interpreted the NIH definition of translational science to be: “a unidirectional continuum in which research findings are moved from the researcher’s bench to the patient’s bedside and community. In the continuum, the first stage of translational research (T1) transfers knowledge from basic research to clinical research, while the second stage (T2) transfers findings from clinical studies or clinical trials to practice settings and communities, where the findings improve health” [23]. It is clear that elements of clinical (and basic) science are undoubtedly present in the definition of translational science, but the NIH’s definition of translational science alludes to a broader translational continuum. It is worth noting that some translational scientists and evaluators feel strongly that the NIH’s definition should also emphasis that translational research exists on a multidirectional continuum [23].

Given the distinction between CTR, we must consider the validity of using the same bibliometrics to evaluate translational research in comparison to clinical research. The use of these metrics has been questioned over the last 15 years with many of the original pitfalls still existing today [24]. One example of a pitfall, regarding a commonly reported metric used to evaluate both fields of research, is the percentage of publications in a top-tier journal. Other examples of such pitfalls include the varying length of time to publication by journal, the types of articles accepted, and the number of references and citations permitted, all of which are factors that influence bibliometric data. Some CTSA evaluators have begun to determine which bibliometrics most appropriately capture translational research in order to accurately evaluate its impact [25,26]. While these studies provide guidance on translational research evaluation, the need to evaluate research from the training perspective, at the scholar-level and program, still remains.

This study was limited in its generalizability as it only focused on K scholars affiliated with a single academic institution. However, in order to assess the performance of KL2 scholars, it was an initial way to evaluate the impact of a CTR training program using a comparison group as an internal control. It should be noted that while there some are similarities between the K08/K23 and KL2 scholar programs, there are numerous differences. The K08/K23 group represented the best control group for this research as the scholars received training at the same institution. Additional limitations on gathering and comparing more granular grant funding information also limited the scope of these results. At this time, only NIH funding was investigated, but future analyses need to consider a variety of grant funding.

Given the results of this study and the 5-year KL2 advantage reported, it is necessary to consider how bibliometrics are interpreted within translational science and the evaluation of CTR training. Over time, differences between translational and clinical bibliometrics must be interpreted with caution and appropriate metrics for translational science must be established. Research must continue to investigate the performance and outcomes of CTR training across the country in order to demonstrate the value of training on the broader scientific community. Future research will expand the current findings to include multi-institutional comparisons of bibliometric outcomes and follow-on funding with a larger and more varied sample of K scholars from different academic health centers.
